# IL-1β impairs retrograde flow of BDNF signaling by attenuating endosome trafficking

**DOI:** 10.1186/s12974-017-0803-z

**Published:** 2017-02-02

**Authors:** Anthony J. Carlos, Liqi Tong, G. Aleph Prieto, Carl W. Cotman

**Affiliations:** 1 Institute for Memory Impairments and Neurological Disorders, University of California Irvine, Irvine, CA 92697 USA; 2 Department of Neurobiology and Behavior, University of California Irvine, Irvine, CA 92697 USA

**Keywords:** IL-1β, BDNF, Endosome trafficking, Erk5

## Abstract

**Background:**

Pro-inflammatory cytokines accumulate in the brain with age and Alzheimer’s disease and can impair neuron health and cognitive function. Brain-derived neurotrophic factor (BDNF) is a key neurotrophin that supports neuron health, function, and synaptic plasticity. The pro-inflammatory cytokine interleukin-1β (IL-1β) impairs BDNF signaling but whether it affects BDNF signaling endosome trafficking has not been studied.

**Methods:**

This study uses an in vitro approach in primary hippocampal neurons to evaluate the effect of IL-1β on BDNF signaling endosome trafficking. Neurons were cultured in microfluidic chambers that separate the environments of the cell body and its axon terminal, enabling us to specifically treat in axon compartments and trace vesicle trafficking in real-time.

**Results:**

We found that IL-1β attenuates BDNF signaling endosomes throughout networks in cultures. In IL-1β-treated cells, overall BDNF endosomal density was decreased, and the colocalization of BDNF endosomes with presynaptic terminals was found to be more than two times higher than in control cultures. Selective IL-1β treatment to the presynaptic compartment in microfluidic chamber attenuated BDNF endosome flux, as measured by reduced BDNF-GFP endosome counts in the somal compartment. Further, IL-1β decreased the BDNF-induced phosphorylation of Erk5, a known BDNF retrograde trafficking target. Mechanistically, the deficiency in trafficking was not due to impaired endocytosis of the BDNF-TrkB complex, or impaired transport rate, since BDNF endosomes traveled at the same rate in both control and IL-1β treatment groups. Among the regulators of presynaptic endosome sorting is the post-translational modification, ubiquitination. In support of this possibility, the IL-1β-mediated suppression of BDNF-induced Erk5 phosphorylation can be rescued by exogenous ubiquitin C-terminal hydrolase L1 (UCH-L1), a deubiquitinating enzyme that regulates ubiquitin and endosomal trafficking.

**Conclusions:**

We observed a state of neurotrophic resistance whereby, in the prolonged presence of IL-1β, BDNF is not effective in delivering long-distance signaling via the retrograde transport of signaling endosomes. Since IL-1β accumulation is an invariant feature across many neurodegenerative diseases, our study suggest that compromised BDNF retrograde transport-dependent signaling may have important implications in neurodegenerative diseases.

## Background

Signaling by BDNF via the TrkB receptor is an important mechanism supporting neuronal survival and activity-dependent plasticity, which is critical for cognitive function [1]. Receptor trafficking, including endocytosis, sorting and transport, is key to BDNF signaling [2, 3]. BDNF follows many of the central tenets of the signaling endosome hypothesis [4]. BDNF binds to its receptor, TrkB, and signal transduction is mediated through the receptor tyrosine kinase domain of the TrkB receptor. The activated complex is propagated to both local and distal subcellular compartments in endosomal vesicles. If the signal initiates on the presynaptic terminal, the vesicle-bound neurotrophins retrogradely traverse intracellular compartments via dynein motors and are regulated by Rab GTPases [5–7]. These endosomes therefore can carry complete signaling complexes capable of activating multiple intracellular cascades leading to plasticity and survival. In cultured neurons, retrograde trafficking of BDNF-signaling endosomes originating in presynaptic compartments activates somal extracellular signal-regulated kinase 5 (Erk5) [8].

Highlighting the significant role endosomal trafficking dysfunction may play in neurological diseases comes from the pathological enlargement of early endosomes in the Alzheimer’s disease (AD) brain, and that many AD-related genes identified as risk factors by genome-wide association studies (GWAS) are involved in different stages of endosomal trafficking (e.g., PICALM, SORL1) [9, 10]. Moreover, disruption of ubiquitin homeostasis, which regulates endosome sorting, is a consistent observation in the pathophysiology associated with Alzheimer’s disease (AD). Post-mortem analyses of AD brains typically exhibit increased levels of ubiquitinated proteins [11] accompanied by inhibition in proteosomal degradation. Of particular interest is a deubiquitinating enzyme, UCH-L1, which is part of a class of deubiquitining enzymes that regulates bioavailability of ubiquitin and is downregulated in AD. In APP/PS1 mice, injection of UCH-L1 improves the retention of contextual learning through the PKA-CREB pathway [12] and reduces Aβ production, increased free ubiquitin and accelerates proteosomal degradation of APP [13]. In our past studies, UCH-L1 rescued the BDNF-Erk5 retrograde trafficking pathway caused by Aβ [14]. In this study, we aimed to extend these findings to interleukin-1β (IL-1β).

IL-1β is a key component of the microglial-mediated immune response in the brain and has deleterious effects on cognition and synaptic plasticity at high or chronic levels of exposure [15, 16]. In the mouse hippocampus, sustained expression of IL-1β impairs contextual and spatial memory [17]. We have reported that IL-1β suppresses long-term potentiation (LTP), the major cellular mechanism of learning and memory, and BDNF-induced Akt, and CREB activation [18–21]; while others have shown a link between acute neuroinflammation and disruption of specific neural circuit functions and cognitive impairment [22]. Studies have also shown that blocking IL-1β signaling can restore cognitive function [20, 23]. We hypothesize that the presence of IL-1β undermines proper endosomal function and compromises BDNF/TrkB signal transduction and synaptic plasticity, placing the brain at risk for cognitive decline and neurodegeneration.

Here, we introduce a new mechanism in which IL-1β fundamentally alters BDNF-TrkB endosomal trafficking, leading to measurable deficits in downstream target Erk5. The nature of the trafficking aberration is not related to vesicle transport rate, but is interrelated to ubiquitin modification, since transduction of UCH-L1 rescues trafficking-dependent downstream effectors. We suggest that in the presence of IL-1β, BDNF-TrkB endosome transport is impaired, leading to downstream signaling deficits.

## Methods

### Primary neuronal cultures and treatments

The use of all animals was approved by the Institutional Animal Care and Use Committee at the University of California, Irvine. Primary neuronal cultures were obtained from embryonic day 18 (E18) rat embryos. Dissociated cells were cultured on either pre-coated poly-L-Lysine 6-well plates (Biocoat plates, BD Bioscience) or poly-L-Lysine coated glass coverslips affixed to microfluidic chambers at 1 × 10^6^ cells/9.5 cm^2^ and 5 × 10^6^ cells/ml (for microfluidic chamber), respectively. All cells were maintained in Neurobasal (Gibco) supplemented with B27, penicillin/streptomycin, and Glutamax at 37 ^o^C, 5% CO_2_, 95% humidity. Rat recombinant IL-1β (PeproTech) was used at a final concentration of 10 ng/ml. Human BDNF (PeproTech) was used at a concentration of 50 ng/ml and Ciliobrevin D (Sigma) was dissolved in sterile Me_2_SO at 100 μM and used at 1 μM.

### Microfluidic chambers

The chambers were fabricated in PDMS using rapid prototyping and soft lithography similar to previously published procedures [24]. Glass coverslips (24 × 40 mm, No. 1, Corning) sonicated in 95% EtOH (30 min) and dried in a sterile hood were immersed in sterile aqueous solution (0.5 mg/ml poly-L-lysine, Sigma) in PBS (24 h, 5% CO_2_, 37 °C incubator), rinsed, and allowed to air dry in a sterile hood. The chambers are noncovalently assembled and remained affixed to glass coverslips by conformal contact. The chambers consist of two parallel microfluidic compartments, connected by inlet and outlet wells. The two compartments are separated by a solid barrier region with microgrooves embedded in the bottom of the connecting barrier. A volume difference between the two compartments of 20 μl is used to generate a fluidic resistance within the microgrooves, facilitating the isolation of treatments.

### Live cell trafficking experiments

Endotoxin-free BDNF-GFP plasmid was introduced by nucleofection (Lonza Amaxa) into HEK293 cells followed by selection in DMEM containing 10% FBS and G418 (1 mg/ml, plasmid contains a neomycin cassette). BDNF-GFP was isolated from stable pre-pro-BDNF-GFP HEK293 lines after cells reached confluency. Secreted BDNF-GFP from the media was removed and concentrated with Amicon YM-30 centrifugal filters (5000 × *g*, 2 h) (30,000 MW cutoff, Millipore). Pro-BDNF was converted to mature BDNF-GFP by treatment with plasmin enzyme (Sigma). Time-lapse microscopy on live cells was utilized to measure the rates of BDNF-GFP-containing endosomes within the microfluidic devices. Prior to treatment, 20% of the cell culture media volume was removed from the axon terminal (treated) side of the chambers. BDNF-GFP or BDNF-GFP + IL-1β were then introduced to the wells. Regions of interest from axon segments from each chamber were randomly selected for time-lapse imaging. After 1 h, transport rates were quantified on an inverted Olympus IX70 with a 63× oil emersion objective. The objective was focused on randomly chosen microgrooves of the chamber and imaged in a time-lapse in 5 s intervals for 1 min. Image stacks from the time-lapse were imported into ImageJ and tracked using ManualTracker. These results were independently verified using GradienTech Particle Tracking Tool Software. For speed measurements, we used the accumulated distance to time ratio. For velocity measurements, we used the Euclidian distance to time ratio.

### Cell surface biotinylation assays

To assess TrkB internalization, primary neurons (7 DIV) were either treated with or without BDNF (50 ng/ml) and then placed on ice to prevent further TrkB internalization. The remaining cell surface TrkB receptors were biotinylated with Sulfo-NHS-LC-Biotin (100 mg/ml, 30 min; Thermo) and then washed with 0.1 M PBS (pH 7.5), three times. The cells were lysed with radioimmunoprecipitation assay buffer containing a protease inhibitor mixture (Roche Applied Science), and biotinylated TrkB was precipitated with streptavidin-agarose beads (Thermo, 50 μl) that had been pre-equilibrated in radioimmunoprecipitation assay buffer. To elute, precipitated proteins were incubated in sample buffer and processed for Western blot analysis.

### Western blot analysis and antibodies

Following boiling or elution in Laemelli Sample Buffer lysates were run on a complete Bio-Rad Western Blotting System. Samples were electrophoretically separated on Bio-Rad 10% Tris-TGX PreCast Gels and blotted onto nitrocellulose membranes using Bio-Rad Trans Turbo Dry transfer and buffer. All blots were blocked using 5% BSA. The following is a list of all primary antibodies used. Phosphorylated-Erk5 (T218/Y220) (1:200, Cell Signaling), ERK5 (1:1000, Cell Signaling), TrkB (1:1000, EMD Millipore). Immunoreactivity was measured using Bio-Rad HRP conjugated secondaries. Blots were developed on a Bio-Rad Digital Detection System running ImageLab software, using Millipore Crescendo chemiluminescence development reagent. ImageJ was used for band densitometry analysis. ImageJ quantification metrics and western blots from the same lysate were ensured to be reasonably reproducible, and all experiments were reproduced using completely independent cultures from different E18 litters.

### Immunocytochemistry and antibodies

Cells were immediately placed on ice and washed gently in ice-cold HBSS. Cells were then fixed in fresh, ice-cold 4% paraformaldehyde for 20 min. The microfluidic chamber devices were removed from the coverslip and cells were fixed again in 4% paraformaldehyde (20 min). Cells were blocked (cold 5% BSA in 0.1 M PBS) and permeablized (0.02% Triton-X100 in 0.1 M PBS) followed by primary antibody, overnight. The following antibodies were used in this study (Anti-GFP 488 conjugate, Life Tech, 1:200; anti-synaptophysin, Millipore, 1:200; Tau-1 (1:200), followed by Alexa conjugated goat-anti-X secondary antibody (Life Tech, 1:200, *X* = primary host). All images were collected using a Leica confocal microscope. Regions of interest [9] were randomly chosen and imaging proceeded serially and sequentially. ImageJ was used to define the number of terminals as the number of defined puncta positive for the synaptophysin marker and to define colocalization consistently across all sections. The output measure was linear density (events per μm) of colocalized events. Axon segments totaling 2445 total terminals were analyzed. For UCHL1 experiments, cell culture medium (40 μl) was removed from each axonal well prior to the addition of BDNF (50 ng/ml) to restrict BDNF to only the axonal compartment. After 90 min treatment, somal compartments were analyzed for phos-Erk5 activation by immunocytochemical analysis as described previously [14]. In brief, the microfluidic devices were removed, and the coverslips were rinsed with PBS, paraformaldehyde-fixed (4%), permeabilized in 0.25% Triton X-100 in PBS (pH 7.4), and blocked with 5% goat serum. The cells were incubated in appropriate primary antibody overnight at 4 °C. phos-Erk5 was stained with anti-phos-Erk5 (1:1000; Cell Signaling) followed by anti-mouse Alexa 488. The cells were washed and then immunolabeled with TO-PRO3 (Invitrogen) to identify nuclei. Images were captured by Zeiss LSM510 two-photon microscope. The mean pixel intensity for each cell was determined and normalized to TO-PRO3.

### Flow cytometry analysis

All the steps for synaptosome P2 fraction isolation were carried out at 4 °C; sucrose buffer, grinder, pestle, and microfuge tubes were all pre-cooled on ice. Rat neuronal cultures >12DIV were gently homogenized in 0.32 M sucrose containing HEPES [10 mM] and protease/phosphatase inhibitors cocktail (Pierce), pH 7.4. The extracts from individual wells, containing 1 × 10^6^ neurons, were pooled. Homogenization consisted of 6–8 manual strokes in a glass-Teflon grinder, clearance (between plunger and glass): 0.15–0.25 mm. Plunger was gently rotated during strokes while the grinder was kept on ice. The extract was first centrifuged at 1000 × *g* for 10 min; the resulting supernatant was centrifuged at 13,000 × *g* for 20 min to obtain the crude P2 pellet, which was resuspended in ice-cold PBS. A P2 fraction aliquot was used to determine protein concentration (BCA assay using BSA as a standard). Synaptosomal P2 fractions were subjected to established immunolabeling protocols to intracellular detection by flow cytometry, as previously described [7, 20, 25], reproduced here, in brief. P2 fractions were fixed in 2% paraformaldehyde and permeabilized in 90% methanol. After labeling, pellets were washed twice and then resuspended in PBS buffer (0.5 ml) for flow cytometry analysis. Anti-Synapsin-1 IgG was labeled directly with Alexa Fluor 647 (Cell Signaling). Background fluorescence thresholds were set by immunolabeling with host-IgG-647 isotype matched control on a non-GFP-treated sample. Comparisons were made from 3 independent pooled samples. Percent gated events positive for both synapsin-1 and GFP were used for the analysis. Samples were acquired using a Becton Dickinson FACSCalibur flow cytometer (BD Biosciences, San Jose, CA, USA) equipped with argon 488 nm and helium-neon 635 nm lasers. Relative size and granularity was determined by forward (FSC) and side scatter (SSC) properties. FSC, SSC, and fluorescence (FL1 [530 ± 15 nm] and FL4 [650 ± 25 nm]) signals were collected using log amplification. FSC-SSC plots were used to select particles matching the size of synaptosomes (0.75–3.0 μm) using calibrated beads (Polysciences, Inc.) as previously described [20, 25]. Identical FSC settings were used for acquiring data on bead standards and samples. Small fragments and debris were excluded by establishing a FSC-H threshold (gain =325). Ten thousand size-gated particles were collected and analyzed for each sample; event rate: approximately 500/s. Analysis was performed using the FlowJo v10 software (FlowJo LLC).

## Results

**Fig. 1:**
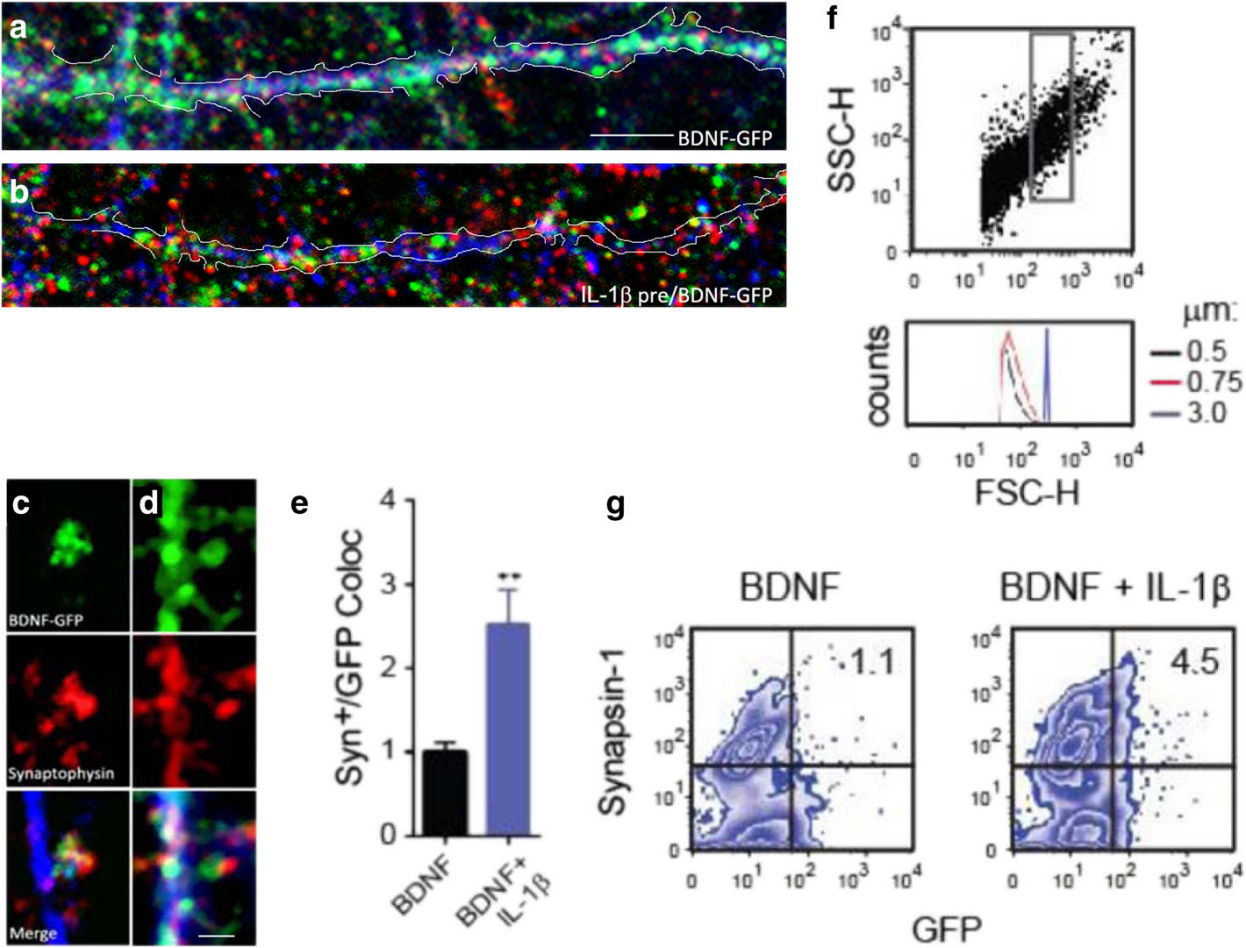
Increased presynaptic colocalization of BDNF-GFP caused by IL-1β as an indicator of reduced endosome transport. Presynaptic colocalization of BDNF-GFP was examined by immunostaining of BDNF-GFP and synaptophysin. Neurons were fixed and analyzed after 1 h post-treatment to assess BDNF-GFP endosome dispersion through neurites (illustrated by traces) in culture. **a**–**d** Representative images that show neurons treated with BDNF-GFP (**a** and **c**) or BDNF-GFP + IL-1β (**b** and **d**) and stained with antibodies against GFP [34], synaptophysin [52], and tau-1 (*blue*). *Scalebars:* 10 μm in **a**–**b** and 2 μm in **c**–**d**. **e** Quantification of the colocalization of BDNF-GFP and synaptophysin in neurons treated with BDNF-GFP or BDNF-GFP + IL-1β. IL-1β treatment was associated with a 2.6-fold increase in BDNF-GFP/synaptophysin colocalization. Graph depicts mean ± SE (as fold relative to BDNF); BDNF = 2.6 ± 0.4, *n* = 3 vs BDNF + IL-1β = 1.0 ± 0.2, *n* = 3 independent cultures. **f** P2 fractions were isolated from three independent cultures and pooled as an alternate means of colocalization. By flow cytometry analysis, forward-Side (FSC-SSC) density plot shows the size-complexity profile of particles in the P2 fraction isolated from hippocampal cultures. The gate (*inside rectangle*) selects according to size (0.75 < gated particles < 3.0 μm), relative to calibrated beads (**f**, *lower panel*) [34, 52]. **g** The population of size-gated synaptosomes was probed for the presence of the presynaptic marker synapsin-1 and BDNF-GFP. The presence of GFP signal in synapsin-1-positive events was used as an indicator BDNF-GFP retained in presynaptic compartments. BDNF + IL-1β groups exhibited a fourfold increase in GFP events colocalized with synapsin-1 (*n* = 3 independent cultures pooled). Thresholds for endogenous/non-specific fluorescence for each marker were set using untreated (no BDNF-GFP) synaptosomal fractions stained with an IgG isotype-matched conjugated to Alexa 647

IL-1β has been previously shown to impair BDNF signaling pathways [18–21]. It is possible that endosomal trafficking of BDNF and signaling pathways activated by BDNF are not independent of each other. The central aim of this study is to define and to begin characterizing the mechanism by which IL-1β affects the trafficking of the BDNF endosomal signaling complex itself. Here, we put forth the evidence as to nature and extent to which IL-1β affects BDNF endosome trafficking. We measured the extent to which BDNF-GFP was found colocalized to presynaptic compartments and found that IL-1β treatment was associated with a 2.6-fold increase in BDNF-GFP colocalization (Fig. 1a–e, *p* < 0.05, Fisher’s exact test), suggesting there was a significant correlation in a controlled experimental culture between IL-1β treatment and localization of these vesicles. To confirm and extend these observations and measure presynaptic BDNF-endosome clustering by alternative method, we isolated P2 synaptosomal fractions (enriched in synaptosomes [26]) from neuron cultures, as previously described [27, 28]. We and others have previously shown that synaptosomes can be identified by flow cytometry using size-calibrated beads [25, 29–31], a strategy based on the analysis of the forward-scattered (FSC) light which is proportional to the size of particles (Fig. 1f). Using our synaptosome immunostaining protocol [31], we examined the population of size-gated synaptosomes that were positive for presynaptic marker synapsin-1 and GFP, as an indication of BDNF-endosome clustering at the presynaptic terminal at the time of collection. By this approach, we found that size-gated synaptosomes positive for both synapsin-1 and BDNF-GFP were increased in cells exposed to IL-1β (Fig. 1g), suggesting increased colocalization of BDNF-GFP at presynaptic terminals by two independent experimental methods.

**Fig. 2:**
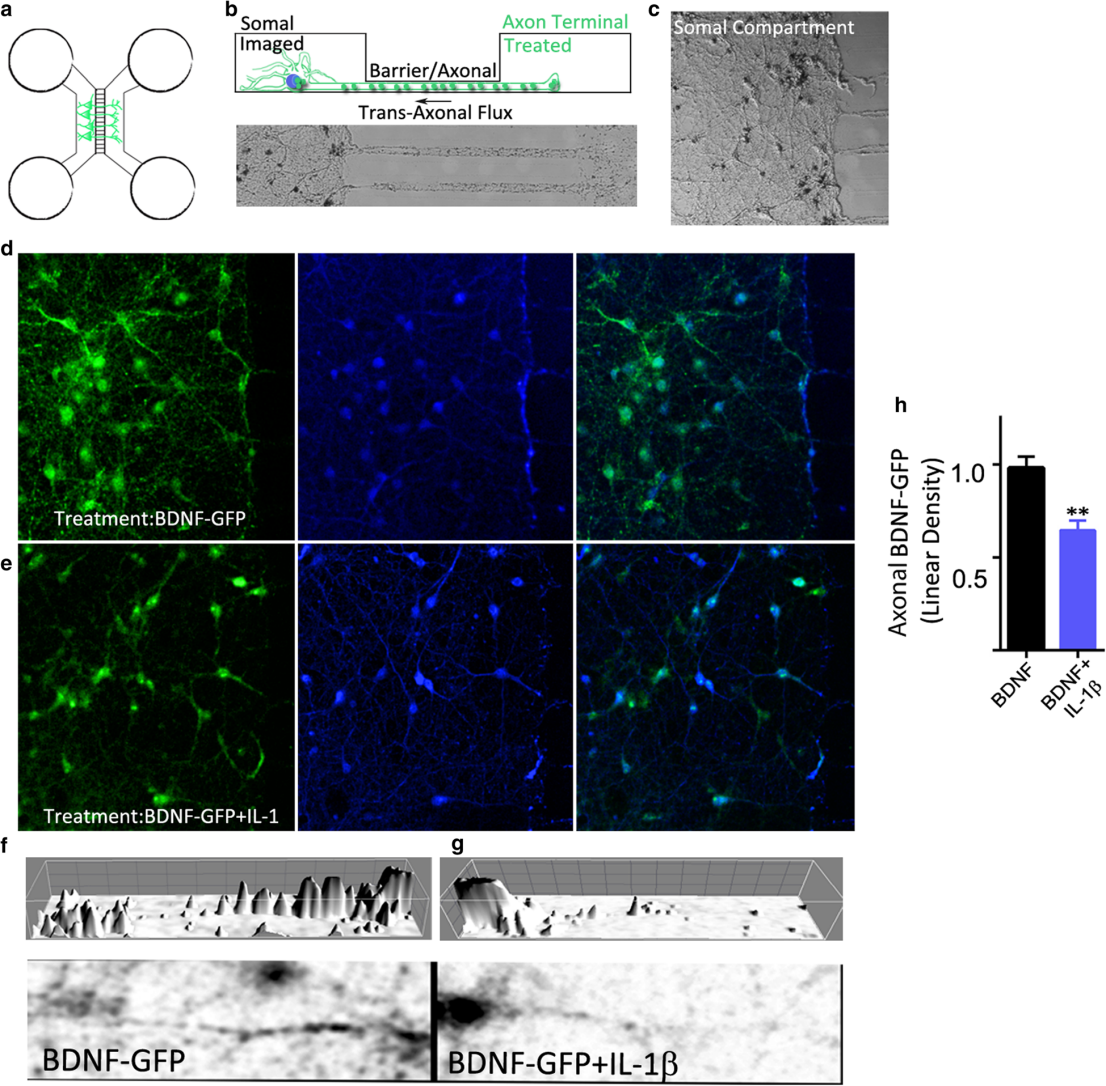
IL-1β reduces trans-axonal long-range BDNF endosome volume flow. **a** Top-down schematic of neurons in microfluidic chambers. **b** Edge on schematic (*upper panel*) and top-down microscope image (*lower panel*) of the three compartments in the microfluidic chambers. **c** Representative neuron culture in microfluidic chambers. **d** and **e** Presynaptic boutons in axon terminal compartment were treated with BDNF-GFP or BDNF-GFP + IL-1β, after which GFP-fluorescence data was collected by analyzing linear density along axons treated in microfluidic chambers. Fluorescence by BDNF-GFP endosomal in the somal compartment was analyzed for as an indicator of BDNF-GFP that arrived via long-range trans-axonal transport mechanisms. Every attempt was made to exclude dendrites from the analysis (e.g., only quantifying along tau-positive segments and only treating the axonal side), thus, everything in the somal side must have arrived via axonal transport. Images are of somal compartments. **f** and **g** Neurites showing a train of BDNF-GFP endosomes in 3D (*upper*) and 2D (*lower*). Three-dimensional images represent intensity peaks and were used for quantification. **h** Quantification of axon-associated BDNF-GFP in soma compartment. BDNF + IL-1β treatment resulted in a 34 ± 9% decline in GFP linear density (unpaired *t* test, *p* < 0.05), relative to BDNF. Mean ± SE; BDNF = 1.0 ± 0.08, *n* = 9 vs BDNF + IL-1β = 0.72 ± 0.08, *n* = 6, unpaired two-tailed *t* test, *p* < 0.05)

Next, we assessed long-range retrograde trafficking using microfluidic chambers we developed that allow the fluidic separation of axons and presynaptic boutons from cell bodies by a distance of 450 μ. This cell culture platform directs the growth of axons from one compartment through a barrier region into an adjacent fluidicially separated target compartment. Thus, any BDNF-GFP introduced to the presynaptic terminal side can only arrive in the somal compartment via long-range intra-axonal retrograde trafficking mechanisms (Fig. 2a–c). We selectively introduced BDNF-GFP or BDNF-GFP + IL-1β to the presynaptic compartment and quantified BDNF-GFP endosomes in the somal compartment 1 h thereafter as a measure of retrograde trafficking. We found that IL-1β treatment reduced BDNF-GFP endosome counts by 29 ± 10% relative to BDNF-GFP-only treatment controls (100 ± 6%, *n* = 9, *p* < 0.05) somal compartments (Fig. 2d–h), suggesting long-range BDNF endosome flux is attenuated by IL-1β.

**Fig. 3:**
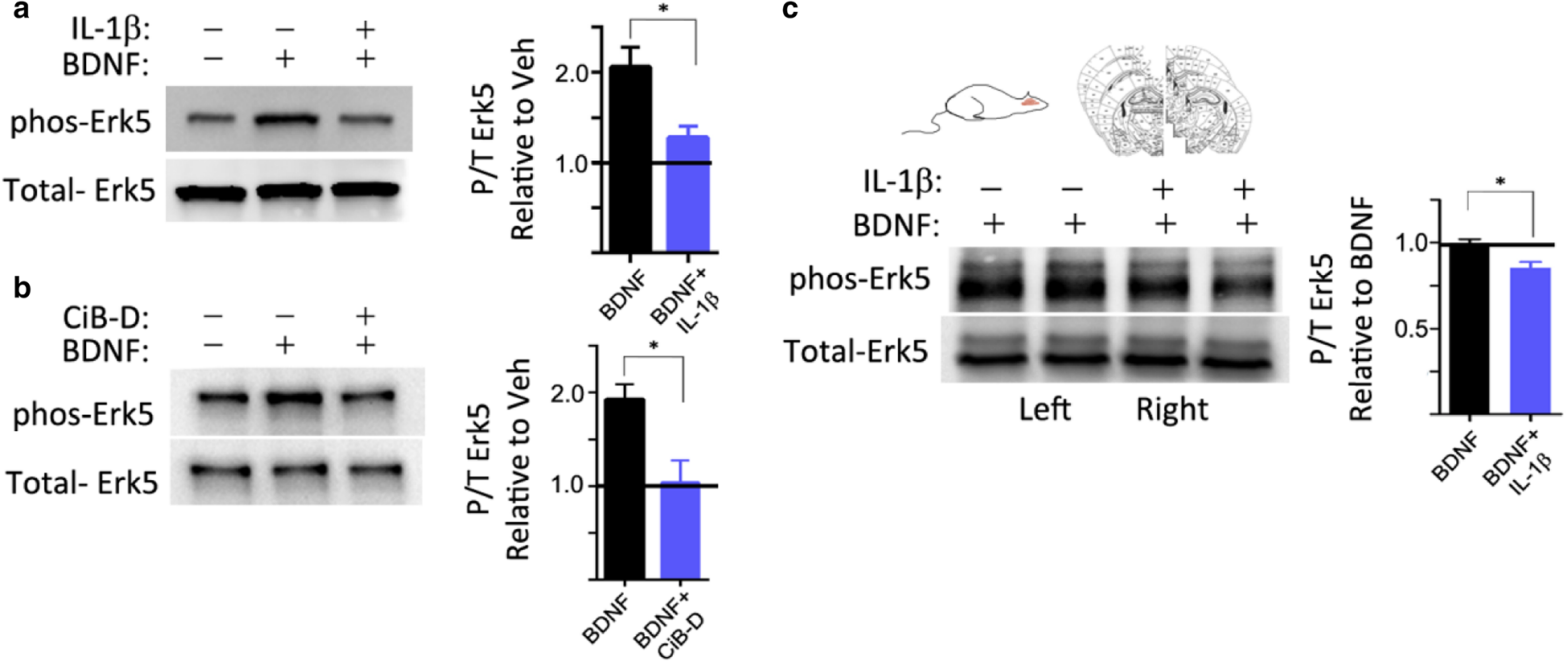
IL-1β attenuates BDNF-induced phosphorylation of retrograde signaling target Erk5. **a** Levels of phos-Erk5 were measured in cultured neurons with BDNF or BDNF + IL-1β treatments. Neurons treated with BDNF exhibited twofold increase in phos-Erk5 levels over baseline non-treated controls while BDNF + IL-1β treatment exhibited only a 1.3-fold increase in phos-Erk5 levels over baseline, suggesting IL-1β attenuates BDNF-induced phosphorylation of Erk5. Mean ± SE; BDNF = 2.0 ± 0.2, *n* = 4 independent cultures vs BDNF + IL-1β = 1.1 ± 0.2, *n* = 3 independent cultures, unpaired two-tailed *t* test, *p* < 0.05). **b** Levels of phos-Erk5 in response to BDNF in the presence of Ciliobrevin D (BDNF + CiB-D)B were reduced to baseline: mean ± SE; BDNF = 1.9 ± 1.4-fold, *n* = 5 independent cultures vs BDNF + CiB-D = 1.1 ± 0.25-fold (by unpaired *t* test *p* < 0.05) **c** Levels of phos-Erk5 in ex vivo slices with BDNF (*left hemisphere*) or BDNF + IL-1β (*right hemisphere*) compared in adult rats that exhibited 14 ± 2% decrease in phos-Erk5 levels after treatment with BDNF + IL-1β when compared to BDNF-only treatment. Graph rescaled such that xBDNF=1.0. Mean ± SE; BDNF = 1.0 ± .02-fold, *n* = 4 animals vs BDNF + IL-1β = 0.84 ± .02-fold, *n* = 4 animals, paired *t* test: *p* < 0.05)

If trafficking deficits are truly associated with IL-1β, then we would expect a long-range BDNF endosome target to also be impaired by IL-1β. Erk5 is a well-established target kinase of BDNF-TrkB endosomes and a central signaling component of gene transcription related to neuronal survival [8, 32]. Evidence has shown that Erk5 becomes phosphorylated in the presence of BDNF and typically demonstrates somal localization and nuclear translocation upon neurotrophin-induced phosphorylation. Erk5 is the first MAP kinase identified whose activity is stimulated by neurotrophins but not by neuronal activity [32]. We therefore tested whether IL-1β could cause downstream signaling deficits to BDNF endosome-induced phosphorylation of Erk5. In cultured neurons, our results indicate that BDNF treatment (50 ng/ml) increases phosphorylated Erk5 (phos-Erk5) 2.1 times baseline (±16%, *n* = 4 independent cultures), while the BDNF + IL-1β group only increased phos-Erk5 levels to 1.2 times baseline (±23%, *n* = 3 independent cultures, *p* < 0.05) (Fig. 3a). To mitigate other possible confounding factors such as BDNF dependent activation of Erk5 via non-trafficking pathways (e.g., somal TrkB, signaling cascades), we inhibited the retrograde trafficking pathway using Ciliobrevin D, a cell-permeable benzoyl dihydroquinazolinone derivative that acts as a reversible and specific blocker of AAA+ ATPase motor cytoplasmic dynein. The remaining BDNF-induced phos-Erk5 would indicate the contribution of non-trafficking dependent phos-ERK5 activation. Figure 3b shows the western blot analysis of phos-Erk5 levels in response to BDNF and BDNF + Ciliobrevin D treatment groups. BDNF-dependent phos-Erk5 increased to 1.8 times baseline (±14%), while Ciliobrevin D attenuated the level of BDNF-induced phos-Erk5, reaching only 1.08 times baseline (±25%, *n* = 5 independent cultures, *p* < 0.05). Since the IL-1β-induced inhibition of trafficking resulted in near baseline levels of phos-Erk5 even in the presence of BDNF, we conclude that there is a functional and measureable consequence of our observations in Figs. 1 and 2. To further extend these findings to a more complex model system, we confirmed that the connection between IL-1β and BDNF-induced phos-Erk5 can also be observed in an acute brain slice assay from 3-month-old rats. Coronal slices prepared from sacrificed rats were treated with recombinant BDNF in the left hemispheres and were compared to BDNF + IL-1β treatments in the animal’s corresponding right hemisphere. By this design, we were able to test both groups, BDNF and BDNF + IL-1β within the same animal. Consistent with the data from our neuron culture experiments, we found that IL-1β reduced BDNF-induced phos-Erk5 14% (*n* = 4, *t* test paired, two tailed, *p* < 0.05) (Fig. 3c).

**Fig. 4:**
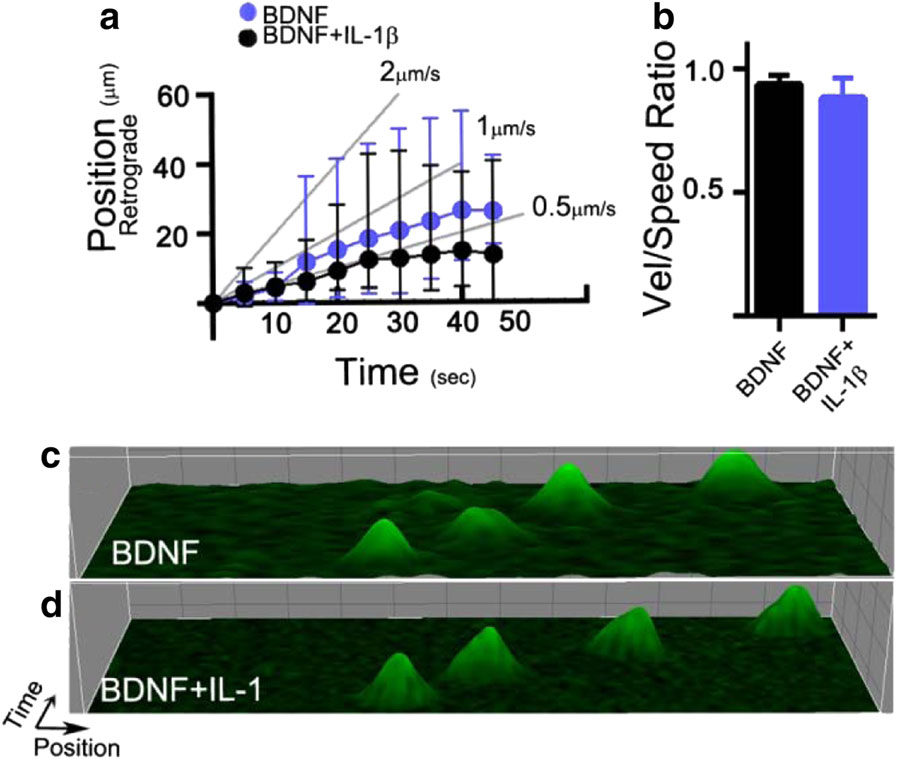
Signaling deficits not associated with a decline in the rate of transport. **a** Displacement vs time of retrograde vesicles in BDNF or BDNF + IL-1β conditions. Endosomes traveled at an average of 0.35 (*n* = 10 cultures used to acquire the data) and 0.5 (*n* = 8) μm/s. Therefore, IL-1β does not reduce the rate of transport of BDNF-GFP vesicles. *Graph* is mean ± range of all values. Transport data were collected from axons running through a single microfluidic groove (<10). **b** Ratio of endosomal velocity to speed, indicating directedness of motion. There is a very minimal difference between the means. Mean ± SE; BDNF = 0.93 ± 0.3, *n* = 10 vs BDNF + IL-1β = 0.87 ± 0.5, *n* = 8. IL-1β does not alter the character of vesicle locomotion. *n* refers to the number of cultures used to acquire the data. **c** 3D kymographs of BDNF and **d** BDNF + IL-1β treatment groups. The 3D kymograph shows the position/time motion of a single vesicle from each group (*X*-axis = position, *Y*-axis = time, *Z*-axis = intensity)

In previous studies, we found that soluble Aβ oligomers (principle neuropathological aggregate of AD) also attenuated BDNF trafficking by reducing the actual rate of transport of BDNF endosomes on axonal microtubules. Since our observations with IL-1β were similar, we asked if there was a common mechanism and if IL-1β affects the rate of transport. We quantified the velocity and speed in live-cell retrograde trafficking experiments to determine if IL-1β-induced endosomal trafficking deficits are directly caused by reduced endosome transport rate. BDNF-GFP particles exhibited a mean rate of 0.35 μm/s (*n* = 10), compared to BDNF-GFP endosomes in cells treated with IL-1β, which exhibited a slightly faster but not significantly different mean rate of 0.5 μm/s (*n* = 8). From these data, we conclude that IL-1β does not reduce BDNF-GFP endosomal trafficking rates (Fig. 4a). To further analyze the character of BDNF endosomal motility, velocity/speed ratios were used as a metric to elucidate the extent to which the endosomal velocity vector was concentrated in actual retrograde motion, as opposed to other observed phenomenon such as stalling, fluttering, or spontaneous reversal (anterograde motion). Since this metric would be sensitive to other motile characteristics, it gives a generalized indication of motile character. Again, we found that the velocity/speed ratios of vesicles in both groups exhibited very similar mean values around 90% retrograde directedness for both groups (Fig. 4b, BDNF: mean ± SEM = 93 ± 3%, *n* = 10, BDNF + IL-1: mean ± SEM = 87 ± 5%), collectively suggesting that neither rates nor transport characteristics are appreciably altered by IL-1β in our cultures.

**Fig. 5:**
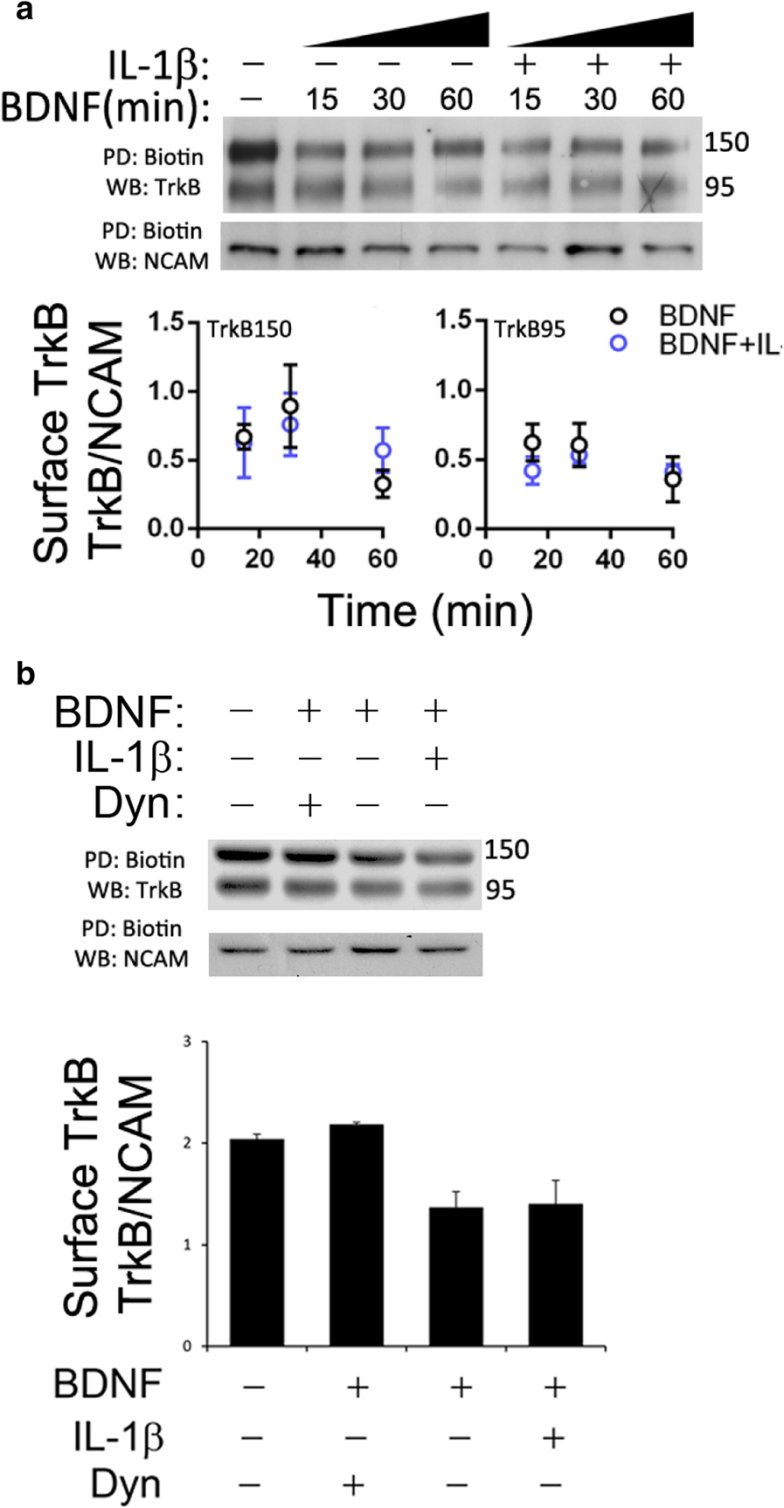
IL-1β does not measurably alter cell surface TrkB expression. **a** Cell surface TrkB was measured in a 1-hour timecourse in response to BDNF or BDNF + IL-1β. Following treatment, endocytosis was arrested and cells were biotinlyated (see Methods). Cell surface TrkB levels did not differ significantly among groups over 1 h. The average rate of endocytosis per 1 h was 53% for TrkB150 after treatment with BDNF vs 47% following treatment with BDNF + IL-1β, suggesting that IL-1β does not significantly alter endocytic kinetics of TrkB at 1 h. Rates ΔCS-TrkB95/hr: BDNF = –72 ± 9%, *n* = 2 independent experiments vs BDNF + IL-1β = –0.70 ± 10%, *n* = 2 independent experiments). Rates ΔCS-TrkB150/hr: BDNF = –72 ± 9%, *n* = 2 independent experiments vs BDNF + IL-1β = –0.70 ± 10%, *n* = 2 independent experiments). **b** Cell surface TrkB-full length (TrkB-FL) was measured in a 1-h timecourse in response to BDNF, BDNF + IL-1β, or BDNF + Dynasore, an inhibitor of dynamin. Dynasore, but not IL-1β, suppressed BDNF-induced TrkB internalization. *Upper panel*: the image of western blotting. *Lower upper panel*: quantification of western blotting data (mean ± SE, *n* = 2)

We next tested whether BDNF-TrkB endocytosis itself was impaired. If less BDNF-GFP endosomes were internalized, it could account for, in part, reduced long-distance BDNF-GFP counts observed in Fig. 2. We used a cell surface biotinylation assay (EZ-Link LC-Biotin, Thermo) to measure whether BDNF-induced TrkB internalization was at all affected by IL-1β over the relevant timecourse of IL-1β treatment. We could find no evidence that IL-1β impaired endocytosis of BDNF (mean ± SE =72 ± 9%, *n* = 2 independent experiments vs BDNF + IL-1β: mean ± SE = 0.70 ± 10%, *n* = 2 independent experiments). In fact, to our surprise, IL-1β may be associated with a slight increase in endocytosis at earlier timepoints (Fig. 5). In the presence of Dynasore (80 μM), an inhibitor of dynamin [33], BDNF-induced TrkB internalization was suppressed (Fig. 5b), supporting the view that IL-1β does not affect BDNF-induced internalization of TrkB. In the absence of altered trafficking kinematics and endocytosis, it follows IL-1β may regulate the egress, or the process of transitioning from presynaptic trafficking mechanisms to long-range axoplasmic trafficking mechanisms. Among regulators of presynaptic endosome, sorting is the post-translational modification, ubiquitination.

**Fig. 6:**
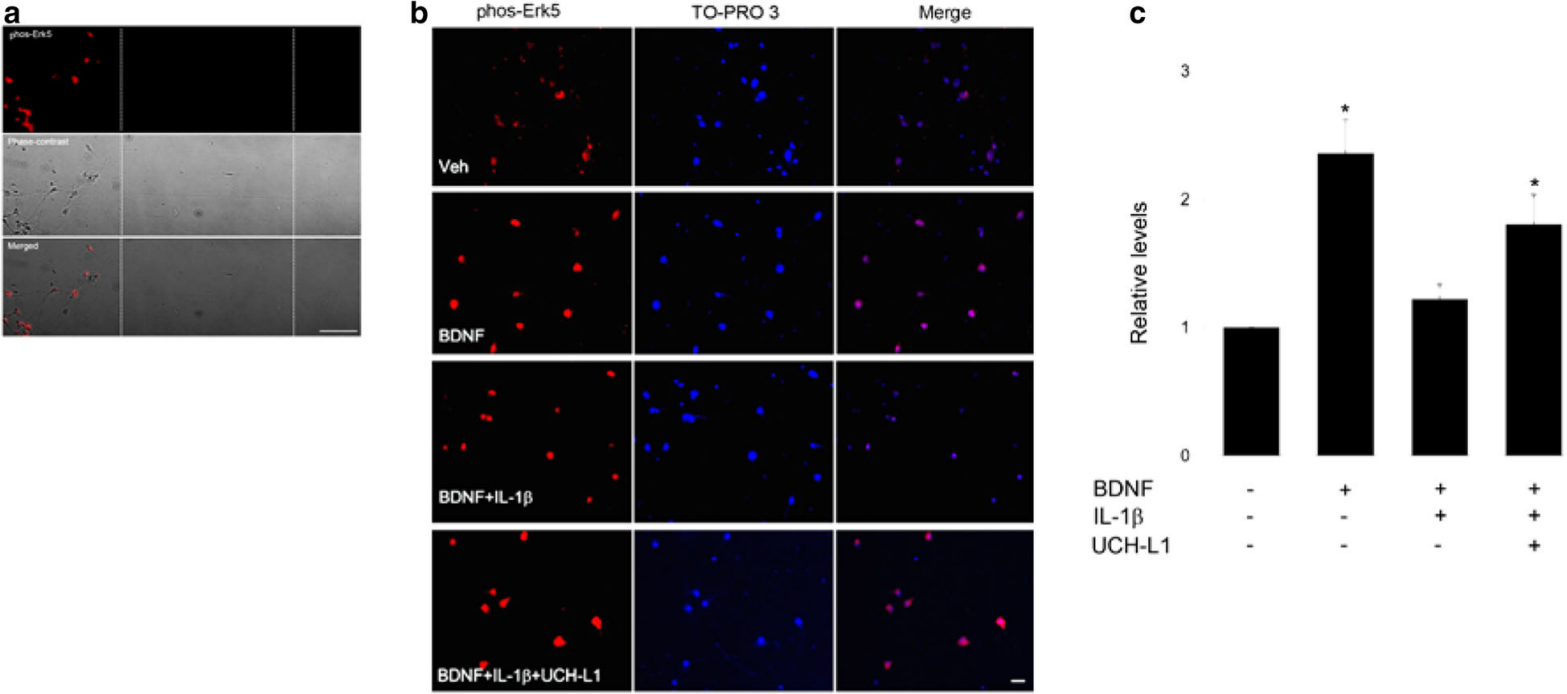
UCH-L1 rescued the IL-1β effect on BDNF-induced Erk5 phosphorylation. phos-Erk5 activation was measured in somal compartment. **a** Low magnification images of neurons immunolabeled with antibody against phos-Erk5 or imaged with phase-contrast filter (*middle*). The images show somal and axonal compartments, which were separated by microgrooves (between the *dash lines*). *Scalebar*, 100 μm. **b** The representative images that demonstrate that BDNF led to an increase in somal phos-Erk5. IL-1β decreased BDNF-induced elevation of somal phos-Erk5 levels. In the presence of UCH-L1, the inhibitory effect of IL-1β was reduced. phos-Erk5 immunoreactivity was normalized to the nuclear counterstain, TO-PRO 3. **c** Quantification of somal phos-Erk5 levels. BDNF treatment significantly increased somal phos-Erk5 levels (*, *p* < 0.05, *n* = 3). In the presence of IL-1β, BDNF treatment did not lead to an elevation of somal phos-Erk5 levels. In the presence of UCH-L1 and IL-1β, BDNF treatment increased somal phos-Erk5 levels (*, *p* < 0.05, *n* = 3). *Scalebar*, 20 μm. *Veh*, vehicle

Because ubiquitin is a widely pervasive mechanism governing synaptic sorting of endosomal cargo, we investigated the effect by introducing a transducible TAT-UCH-L1. UCH-L1 has been shown to restore normal enzymatic activity and synaptic function both in hippocampal slices treated with oligomeric Aβ and in the APP/PS1 mouse model of AD. Based on our previous findings that UCH-L1 rescues BDNF/TrkB retrograde transport deficits induced by Aβ [14], we believe UCH-L1 is a mechanistically interesting and relevant protein of interest. The effect of UCH-L1 on IL-1β-induced impairment of BDNF was examined by measuring BDNF-induced phosphorylation of Erk5 in somal compartment in microfluidic chambers. Like IL-1β and BDNF, TAT-UCH-L1 was applied distally to axon terminals in a distinct fluidic environment from cell somas, which resided in a somal compartment that was 450 μm across a fluid barrier. As shown in Fig. 6, BDNF treatment in the axonal compartment increases phosphorylation of phos-Erk5 in somal compartment 2.4 times baseline ± 26%, *n* = 3 independent cultures), while the BDNF + IL-1β group only increased phos-Erk5 levels to 1.2 times baseline (±12%, *n* = 3 independent cultures). Importantly, UCH-L1 treatment rescued IL-1β-induced impairment of BDNF retrograde signaling to 1.8 times baseline ± 23%, *n* = 3 independent cultures). Our data indicate that IL-1β interferes with pathways that are associated with ubiquitin homeostasis or can at least be, in part, regulated by ubiquitin.

## Discussion

In the current study, we demonstrate that IL-1β impairs BDNF retrograde trafficking. We show that neurons cultured in microfluidic chambers, which fluidically isolate presynaptic terminals from cell bodies, exhibit sustained long-range retrograde transport deficits in the presence of IL-1β. The cytokine-induced long-range trafficking deficit was also evidenced by the inhibition of phos-Erk5, the BDNF endosome retrograde trafficking target. The trafficking defect within the presynaptic compartment was confirmed by synaptosome isolation from cultured hippocampal neurons after IL-1β treatment and immunostaining for BDNF-GFP. IL-1β treatment increased the size-gated events positive for both synaptophysin and BDNF-GFP, confirming increased colocalization of BDNF-GFP at presynaptic terminals.

The BDNF-induced Erk5 activation, which is impaired by Aβ in our previous study [14] and by IL-1β in our current study, has important functional implications for neuronal survival, neurogenesis, and hippocampal-dependent learning and memory. Erk5 mediates neurotrophin-induced changes in gene expression necessary for neuronal survival, notably upregulation of bcl-w, an anti-apoptotic bcl-2 family member [32, 34]. In addition, Erk5 can be activated by neurotrophins in adult neural stem/progenitor cells, promoting neurogenesis in the adult brain [35, 36]. Notably, genetic activation of ERK5 increases adult neurogenesis in the dentate gyrus and improves spatial learning and long-term memory persistence [37]. Taken together, the literature and our data suggest that IL-1β-induced impairment of BDNF-dependent Erk5 action may contribute to the deleterious role of inflammation in aging and neurodegenerative diseases. Previously, we reported that Aβ applied to axons in microfluidic chambers impaired retrograde flow and resulted in long-range BDNF transport deficits, leading to suppression of downstream Erk5 activity [14]. Consistent with our previous study on Aβ [14], IL-1β decreased BDNF-retrograde transport-dependent Erk5 activity in hippocampal neuronal cultures and reduced BDNF-dependent phosphorylation of Erk5 in adult brain slices. It is noteworthy that the inhibition of Erk5 activation was found to be more evident in pure neuronal cultures than in slices, thus, suggesting that the mechanism underlying BDNF-ERK5-IL-1β interactions is, basically, neuron-specific. Indeed, we have recently described that IL-1β suppresses activity-dependent signaling directly at the synapse [25]. Importantly, Aβ was associated with a reduced rate of BDNF endosomal transport, IL-1β did not affect the rate of BDNF/TrkB endosome transport, suggesting that Aβ and IL-1β act via distinct mechanisms and may be able to act synergistically to impair the retrograde trafficking pathway. A tempting but simplified model for AD may rely on a reduced rate of endosomal transport in neurons by early accumulation of Aβ, which also promotes a rise in brain IL-1β levels [38]. According to our data, Aβ and IL-1β may synergistically impair the retrograde trafficking pathway (via different mechanisms), thus, contributing to cognitive impairment in late AD stages.

Our data, that IL-1β has no significant effect on TrkB/BDNF endocytosis or actual transport rates of BDNF/TrkB endosomes, suggest that the deficit in BDNF retrograde trafficking likely stems from impaired endosomal trafficking from the presynaptic terminals and endosome loading onto the retrograde transport system. Potential contributing mechanisms underlying the deficit may include alterations to trafficking accessory protein complexes (such as Rab/Rac family), changes at the level of retromer complexes, actin dynamics, or altered regulation of ubiquitination. Any of these mechanisms would lead to accumulation of BDNF endosomes in presynaptic terminals. Importantly, ubiquitination has been shown to play a critical role in delivery of receptors to multivesicular endosomes (MVBs), the major sorting platform for membrane proteins including trophic factor receptors [39, 40]. Thus, altered regulation of ubiquitination may contribute to IL-1β-induced deficit in endosome loading. It is also possible that regulation of ubiquitination affects other molecules that play key role in BDNF retrograde transport including dynein [41, 42] and Pincher [43]. It remains for future research to explicitly identify the molecular mechanisms underlying IL-1β-induced impairment in BDNF retrograde trafficking.

Disruption of actin dynamics may be a particularly relevant mechanism, given that actin dynamics have been shown to play a key role in the regulation of early endosome dynamics. For example, in Caenorhabditis elegans, disruption of the Arp2/3 complex (which regulates branched actin dynamics) led to significantly larger early endosomes but did not affect endocytosis [44]. In addition, our previous studies have demonstrated that IL-1β impairs BDNF signaling [18, 20, 21] by targeting actin polymerization [18], and that IL-1β impairs BDNF dependent LTP by preventing F-actin formation and spine stabilization [18].

We previously demonstrated that impaired retrograde transport in the presence of Aβ occurs through an ubiquitin-dependent mechanism, and that the deubiquiting enzyme UCHL-1 restores retrograde transport of BDNF/TrkB [14]. Here, we investigated if there is a similar role for ubiquitination in the retrograde transport deficit induced by IL-1β, by introducing a transducible TAT-UCHL-1 construct. We found that UCHL-1 restored BDNF/TrkB transport not only in the presence of Aβ but also with IL-1β, suggesting the two agents share a common mechanism of interfering with ubiquitination-dependent trafficking mechanisms. In support of our data, previous studies demonstrated that ubiquitination regulates Trk receptors endocytic trafficking to multivesicular bodies for sustained retrograde signaling [40, 43, 45]. Thus, the IL-1β induced impairment of ubiquitin homeostasis may exacerbate the suppressive effect of Aβ on endosomal trafficking and synaptic impairments.

Our results support previous studies that have reported disrupted vesicle transport in AD, one of the earliest pathological features in the AD brain and in animal and cell-based models of AD [46, 47]. These studies have shown early endocytic changes including an increased volume of early endosomes as an early stage of several neurological diseases, including AD and Down syndrome [48, 49]. In addition, in amyotrophic lateral sclerosis, endosomes accumulate APP in motor neurons reflecting impaired vesicle trafficking [50], while in Parkinson’s disease, α-synuclein multimers cause synaptic vesicles to cluster and traffick to be attenuated [51]. Taken in context with our findings, neuropathology and chronically upregulated IL-1β may act additively or even synergistically to incapacitate the steady-state vesicle flow throughout the cell and impair neuronal function in various neurodegenerative diseases.

## Conclusion

Our findings extend previous data on the role of IL-1β in neuronal function and show that IL-1β can impair endosomal trafficking and contribute to synaptic and neuronal dysfunction. Endosomal dysfunction can be interrelated to a number of signaling deficits. Because ligand-activated signaling cascades depend on ligand-receptor activation, and activated receptors are distributed intracellularly via endosome transport, it follows that proper endosomal transport is a vital component of many signaling cascades. Endosomal transport dysfunction is a cell-wide problem, since virtually all classes of cell-surface receptors that undergo endocytosis, recycling, or sorting depend heavily on proper endosome trafficking. It is likely that IL-1β impairs endosomal trafficking for other neurotrophic factors besides BDNF, a topic that warrants further investigation.

## Abbreviations

AD: Alzheimer’s disease
BDNF: Brain-derived neurotrophic factor
Erk5: Extracellular signal-regulated kinase 5
GWAS: Genome-wide association studies
IL-1β: Interleukin-1β
LTP: Long-term potentiation
UCH-L1: Ubiquitin C-terminal hydrolase L1
